# Spinal posture, mobility, and position sense in adolescents with chest wall deformities: a comparison of pectus excavatum, pectus carinatum and healthy peers

**DOI:** 10.1007/s00383-024-05759-0

**Published:** 2024-07-06

**Authors:** Oguzhan METE, Hakan IŞIK, Cansu ŞAHBAZ PİRİNÇÇİ, Mustafa Ertuğrul YAŞA, Ersin SAPMAZ

**Affiliations:** 1 Cardiopulmonary Physiotherapy and Rehabilitation Department, Gülhane Faculty of Physiotherapy and Rehabilitation, University of Health Sciences, Ankara, Turkey; 2 Department of Thoracic Surgery, Gülhane Training and Research Hospital, Ankara, Turkey

**Keywords:** Pectus carinatum, Pectus excavatum, Posture, Proprioception, Spine, Thoracic surgery

## Abstract

**Purpose:**

The study aimed to compare spinal posture, mobility, and position sense in adolescents with pectus excavatum (PE), pectus carinatum (PC), and healthy control (HC).

**Methods:**

22 with PE, 22 with PC, and 21 HC were included in the study. The spinal posture (thoracic kyphosis, lumbar lordosis, pelvic tilt, thoracic, lumbar, pelvic lateral tilt angles) and mobility (thoracic, lumbar, hip/sacral, and overall, in the sagittal and frontal plane) with the spinal mouse, and spinal position sense (repositing errors) with the inclinometer were assessed.

**Results:**

The thoracic kyphosis angle of PE and PC was higher than in HC (*p* < 0.001; *p* = 0.001). Hip/sacral mobility in the sagittal plane was lower in the PE and PC than control, respectively (*p* < 0.001; *p* < 0.001). Overall sagittal spinal mobility (*p*:0.007) and hip/sacral mobility in the frontal plane (*p*:0.002) were lower in the PC than in HC. Overall frontal spinal mobility was lower in the PE and PC than in HC (*p*:0.002; *p*:0.014). The PE and PC repositing errors were higher (*p* < 0.001; *p*:0.014).

**Conclusion:**

The study found that adolescents with PE and PC had decreased spinal mobility, spinal alignment disorders, and a decline in spinal position sense. It is important not to overlook the spine during physical examinations of adolescents with chest wall deformities. In clinical practice, we suggest that adolescents with chest deformities should undergo a spine evaluation and be referred for physical therapy to manage spinal disorders.

## Highlights

- Adolescents with pectus excavatum and carinatum had spinal alignment disorders such as increased thoracic kyphosis and lumbar lateral tilt.
- The spinal mobility was reduced in adolescents with pectus excavatum and carinatum.
- Adolescents with pectus excavatum and carinatum had weaker spinal position sense than healthy.
•Adolescents with pectus excavatum and carinatum should undergo a spine evaluation and be referred for physical therapy.

## Introduction

Congenital chest wall deformities are anomalies caused by the absence, shortness, or fusion of one or more costae or cartilages and affect approximately 1% of the population [1, 2]. Pectus excavatum (PE) and pectus carinatum (PC) are the most commonly encountered chest wall deformities in clinical practice [2, 3]. PE is characterized by a depressed sternum and inward displacement of the costal cartilage. It may be present at birth or develop during the first year of life. It usually shows a tendency to progress and most of the time the deformity stabilizes by taking its final shape until adolescence [1, 3]. Depending on the severity of the deformity, cardiac and respiratory problems, cosmetic, psychological, and musculoskeletal problems may occur [4, 5]. PC is characterized by the protrusion of the anterior chest wall. Although the deformity is usually recognized at birth, most of them become more apparent at the beginning of adolescence. Congenital heart diseases, musculoskeletal system defects, and psychological problems may be observed with PC [3, 6].

The rib cage is a bony structure in the chest that serves the crucial function of protecting vital organs. In addition to its protective role, the rib cage, which comprises the sternum, ribs, interconnecting joints, and soft tissue, is also important for spinal biomechanics. It provides a strong framework for the attachment of spinal and abdominal muscles and supports spinal stability and motion through muscular and ligamentous stabilization [7, 8]. To keep spinal alignment and mobility in balance, the geometry and kinematics of the rib cage are important [9]. Therefore, the rib cage acts as a fourth spinal column, especially in the thoracic section [8] and it may be seen as a new element in the spinopelvic chain [9]. Based on this conception, alteration in rib cage anatomic position and biomechanics like in chest wall deformities may result in disorders of spinal alignment and biomechanics [7, 10, 11]. The high incidence of scoliosis, one of the main alignment problems of the spine in PE and PC, was reported [12].

As previously mentioned, while the chest wall plays an important role in the biomechanics of the spine, the assessment of the spine in chest wall deformities is often overlooked. Although previous studies have reported postural disorders, such as hyperkyphosis and scoliosis, in chest wall deformities, they have generally focused on a general postural assessment using observational or scale-based methods [13, 14]. Besides, radiographic studies generally focused on relationships between PE and kyphoscoliosis [12]. To the best of the author’s knowledge, no study has been conducted to evaluate segmental spinal postural alignment in sagittal and frontal planes using three-dimensional imaging in patients with PE and PC. Additionally, no study has investigated spinal mobility and spinal position sense in individuals with PE and PC. Given the close relationship between the rib cage and spine, it is hypothesized that changes in rib cage biomechanics, especially the position of the sternum, may cause disorders in spinal biomechanics, function, and proprioceptive accuracy. Therefore, this study aimed to compare spinal posture, mobility, and position sense in individuals with PE, PC, and healthy peers.

## Materials and methods

### Study design

The study was designed as a single-center, case–controlled study. The study was conducted at Health Science University/Gülhane Training and Research Hospital, Thoracic Surgery Clinic between May 2023 and March 2024. The experimental protocol was approved by the Gülhane Scientific Research Ethics Committee (Protocol No: 2023/134) and this study was performed strictly under the approved guidelines. Written informed consent was obtained from parents. Before scheduling participation, each research step was explained verbally, and an information sheet about the study was given. Participants were allowed to withdraw from the study at any point.

### Participants

Adolescent patients with chest wall deformities and age, body mass index (BMI), and sex-matched healthy controls were included in this study. The patients were divided into two groups according to the type of chest wall deformity (pectus carinatum and pectus excavatum). Healthy controls were recruited among the community via advertisement by modified snowball sampling. A total of three study groups were formed PE, PC, and healthy control.

The study included patients aged between 10 and 17 who were diagnosed with anterior chest wall deformity according to the Willital classification by a chest surgeon [15]. Patients were excluded from the study in case of (a) any chest deformity other than PE and PC such as Poland syndrome, sternal defects etc. (b) having traumatic and iatrogenic chest deformity, (c) any disease that can influence spinal and thoracic cage posture, (d) history of spinal or chest surgery, (e) having a communication problem that cannot comply with the measurements and surveys in study (f) having a sensorial neurologic disease that can affect proprioception (position sense), (g) additional physical problem such as unequal leg length, excessive postural kyphosis, scoliosis, etc., and, (h) not aged between 10 and 17. Age, sex, BMI, and sex-matched healthy controls were included in the study. The same exclusion criteria plus not having any chest wall deformities were applied to the control group.

### Measurements

The demographic characteristics of the participants (sex, age, height, weight, and BMI), disease-related features of participants with pectus deformities (family history of pectus deformities, duration of diagnosis, the reason for applying to the physician (aesthetic, pain, shortness of breath, posture disorder, consultation), history of shortness of breath and fainting/dizziness at rest and during exercise, pain absence and location (no pain, chest pain, back pain) were questioned and recorded on a standard semi-structured form. The spinal posture, mobility, and position sense were measured.

#### Spinal posture and mobility

A spinal mouse (IDIAG M360, Fehraltorf, Switzerland) was used to determine spinal posture and spinal mobility. Three-dimensional spinal posture and mobility can be easily and non-invasively measured by the Spinal Mouse. It provides reporting segmentally and regionally as thoracic, lumbar, and hip/sacral in the sagittal and frontal plane via its software program. It is a reliable device for assessing spinal posture and mobility. Previously reported that it had excellent reliability with intra-tester intraclass correlation coefficients (ICCs) between 0.947 and 0.980, inter-tester (ICCs: 0.949–0.986), and high test–retest (ICCs 0.719–0.908) [16].

**Fig. 1:**
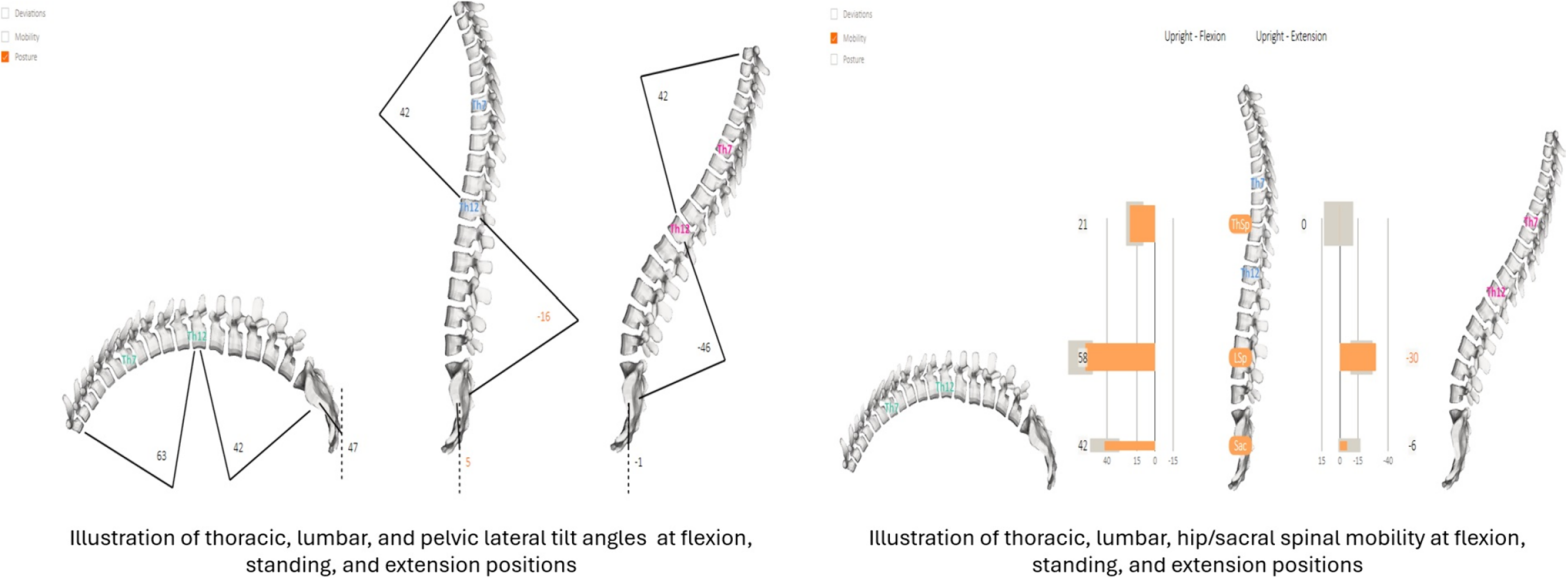
Example of spinal mouse output

The operating procedure was explained to each participant before measurement and the patients’ clothing above their waists was to be removed. Participants were asked to stand in a comfortable standing posture with equal weight bearing on each leg with bare feet. Each vertebral spinous processes were palpated and marked with a cosmetic pen. For the posture assessment, the spinal mouse was placed on the C7 spinous process and moved down steadily to S3 in the standing position. For the spinal posture, spinal curves thoracic kyphosis, lumbar lordosis, and pelvic tilt angles in the sagittal plane, and thoracic, lumbar, and pelvic lateral tilt angles were calculated via the software [17]. For the mobility assessments, two positions were used in the sagittal plane (maximum trunk flexion – maximum trunk extension) and two positions in the frontal plane (maximum trunk lateral flexion to both sides). The difference between maximum flexion and maximum extension was recorded as the sagittal mobility and the difference between left and right lateral flexion was recorded as frontal mobility in degrees. Thoracic, lumbar, hip/sacral and overall spinal mobility in both planes was obtained via the software [17]. The output data are illustrated in Fig. 1.

#### Spinal position sense

A digital inclinometer (Dualer IQ Pro^™^ Digital Inclinometer, JTECH Medical) was used to perform the target angle test referring to position sense (proprioception) was used. The measurement was taken when the participants were sitting flat on a backless chair. The inclinometer was placed on the spinous process of the 4th thoracic vertebra. Before the measurement, the participants were taught the movement of flat sitting to 30^0^ trunk bending position with their eyes closed. Measurement was started after making sure that the patient understood the task and memorized the target angle. The participants were then asked to perform the previously achieved position three times consecutively. The absolute difference in degrees between the target angle (30^0^ trunk flexion) and the participants’ performance angle was recorded as a repositing error. The mean of the three results of each repositing error was recorded as the target angle test score (spinal position sense). The higher repositing error indicates a weaker position sense [11].

### Statistical analysis

The study’s required sample size was calculated using a statistical power analysis software program (G*Power Version 3.0.10, developed by Franz Faul at the University of Kiel, Germany). The size of the sample was estimated based on the sagittal total spinal mobility, and four randomly selected data from each group were used for calculations. The calculations indicated that a sample of 51 participants (17 per group) would be necessary to obtain 80% power, with an effect size of *f* = 0.479, a type I error of *α* = 0.05, a type II error of *β* = 0.20, and a 10% drop rate.

Data analysis and calculations were performed with statistical analysis software (IBM Corp. Released in 2012, IBM SPSS Statistics for Windows, Version 22.0). To examine the distribution of data the Shapiro-Wilks Test, histogram, detrended normal Q-Q graph, skewness and kurtosis coefficients, and coefficient of variation were employed. Continuous values that follow a normal distribution were represented as X ± SD (standard deviation), while those that did not follow a normal distribution were represented as median (Interquartile Range). Categorical variables were represented as frequency (*n*) and percentage (%). The Chi-square Test was used to compare categorical variables. In the case of continuous variables, the one-way ANOVA (Analysis of Variance) test was employed for comparing the variables of different groups, provided that the assumption of normal distribution and homogenous variances were met. If the assumption of normal distribution was tenable but the variances were not homogeneous, the Welch ANOVA test was used. The Kruskal–Wallis test was used when the assumption of normal distribution was not met. Post hoc tests were performed with the Games-Howell and Bonferroni correction methods. Bonferroni correction was carried out after one-way ANOVA and The Kruskal–Wallis test in the presence of significant results. Games-Howell was carried out after The Welch ANOVA test. A result was considered statistically significant if the overall *p*-value was less than 0.05.

## Results

**Fig. 2:**
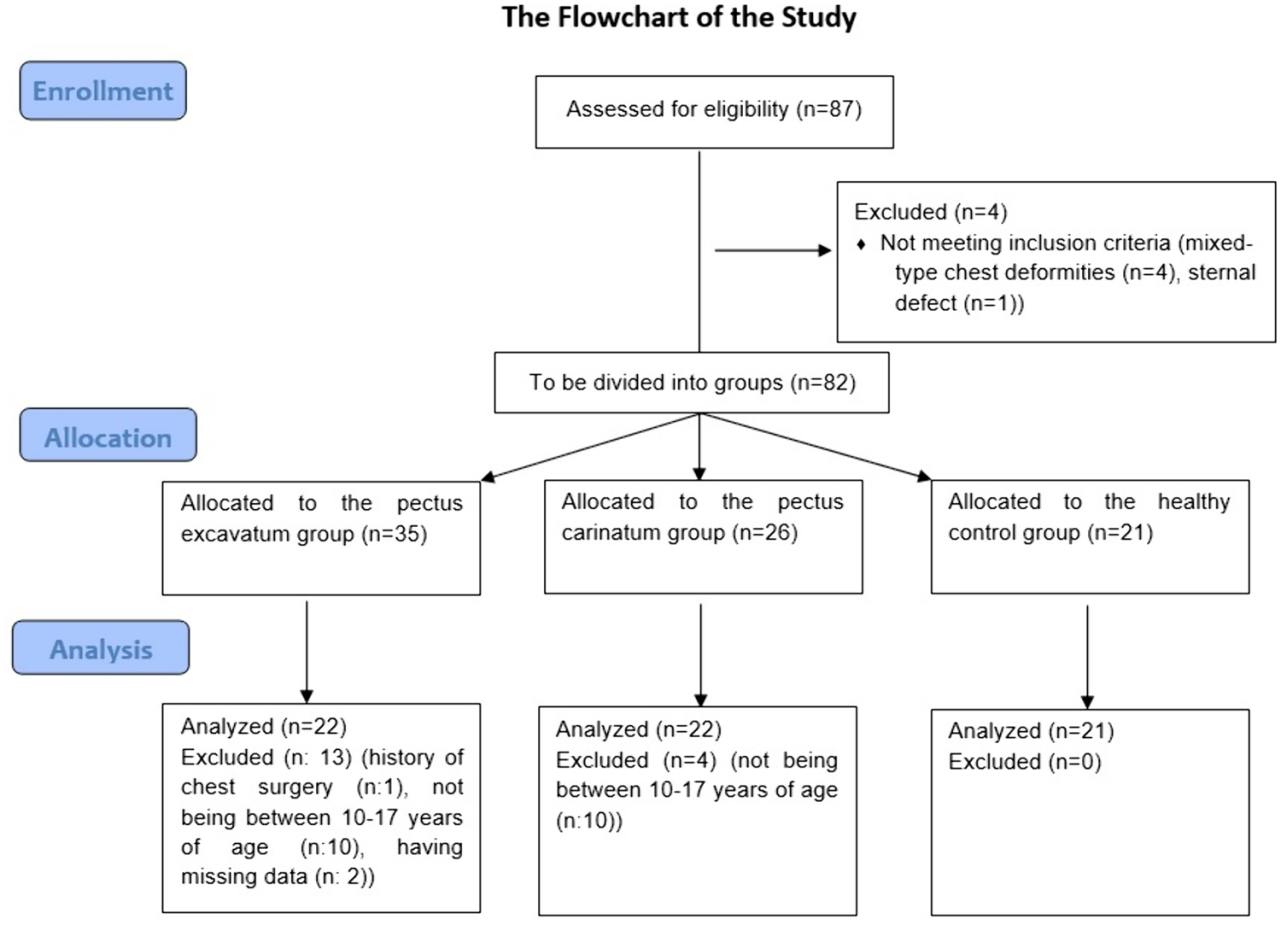
The flowchart of the study

Sixty-six participants with diagnosed chest deformities were assessed by a chest surgeon to determine eligibility criteria. 4 participants with mixed-type chest deformities and one with sternal defects were excluded from the study. Among the remaining, 35 had PE deformities and 26 had PC deformities. 13 participants with PE deformities were excluded from the study due to a history of chest surgery (*n*:1), not being between 10 and 17 years of age (*n*:10) or having missing data in the spinal posture and mobility assessment (*n*:2). 4 participants with PC deformities were excluded from the study for not being between 10 and 17 years of age (*n*:4). The study ultimately included 22 participants with PE deformities, 22 participants with PC deformities, and 21 healthy controls who were matched in sex, age, and BMI, totally 65 participants. A flowchart illustrating the process from assessing eligibility criteria to data analysis for the study is provided in Fig. 2.

The age (*p*:0.110), body height (*p*:0.101), weight (*p*:0.089), BMI (*p*:0.089), and sex (*p*:0.737) of the groups were similar (Table 1). The disease-related features of participants with pectus deformities are given in Table 2.

**Table 1:** Comparison of the demographic and physical characteristics of participants

	Pectus excavatum group (*n*:22)	Pectus carinatum group (*n*:22)	Healthy control group (*n*:21)	*p*
Age (years, mean ± SD)	13.59 ± 2.26	13.72 ± 1.35	12.80 ± 1.63	0.140^a^
Body height (cm, mean ± SD)	164.27 ± 13.11	168.14 ± 13.10	159.61 ± 11.77	0.101^b^
Body weight (kg, median (IQR))	45.50 (16.0)	57.0 (10.50)	50.0 (19.0)	0.089^c^
BMI (kg/m^2^, median (IQR))	17.03 (2.79)	19.53 (1.89)	18.33 (6.29)	0.089^c^
Sex (*n* (%))				
Boy	21 (95.4)	21 (95.4)	19 (90.4)	0.737^d^
Girl	1 (5.6)	1 (5.6)	2 (9.6)	

G1: pectus excavatum group, G2: pectus carinatum group, G3: healthy control groups, P0: comparison of all groups, P1: comparison of pectus excavatum and carinatum groups, P2: comparison of pectus excavatum and healthy control groups, P3: comparison of pectus carinatum and healthy control groups

*SD* standart deviation, *IQR* ınterquartile range, *n* number, *BMI* body mass index

^a^One-way ANOVA, ^b^Welch’s ANOVA, ^c^Kruskal Wallis test, ^d^Chi-Square

**Table 2:** Disease-related features of participants with pectus excavatum and carinatum

	Pectus excavatum group (*n*:22)	Pectus carinatum group (*n*:22)
Duration of diagnosis (months, mean ± SD)	5.95 ± 9,74	4.13 ± 8.32
The main reason for applying to the physician (*n* (%))		
Aesthetic	15 (68.2)	17 (77.3)
Pain	1 (4.5)	0 (0)
Shortness of breath	0 (0)	0 (0)
Posture disorders	3 (13.6)	4 (18.2)
Consultation	3 (13.6)	1 (4.5)
Family history of pectus deformities (*n* (%))		
Yes	8 (36.4)	9 (40.9)
No	14 (63.6)	13 (59.1)
History of shortness of breath (*n* (%))		
At rest		
Yes	1 (4.5)	2 (9.1)
No	21 (95.5)	20 (90.1)
During exercise		
Yes	1 (4.5)	2 (9.1)
No	21 (95.5)	20 (90.1)
History of fainting/dizziness (*n* (%))		
At rest		
Yes	0 (0)	0 (0)
No	22 (100)	22 (100)
During exercise		
Yes	0 (0)	0 (0)
No	22 (100)	22 (100)
Pain (*n* (%))		
No pain	19 (86.4)	19 (86.4)
Chest pain	1 (4.5)	1 (4.5)
Back pain	2 (9.1)	2 (9.1)

*SD* standart deviation, *n* number

**Table 3:** Comparison of spinal posture, mobility, and position sense of groups

Spinal assessment parameters	Measurements	Pectus excavatum (G1)	Pectus carinatum (G2)	Healthy control (G3)	P0	P1	P2	P3
Spinal position sense (Degrees)	Repositing error (mean ± SD)	5.56 ± 1.98	4.18 ± 1.97	2.58 ± 1.32	< 0.001^a^*	0.580	< 0.001** **G1 > G3**	0.014** **G2 > G3**
Sagittal plane spinal posture (Degrees)	Thoracic Kyphosis (mean ± SD)	45.66 ± 11.70	45.54 ± 13.55	32.38 ± 6.28	< 0.001^b^*	0.999	< 0.001** **G1 > G3**	0.001** **G2 > G3**
	Lumbar lordosis (mean ± SD)	-8.80 ± 27.32	-14.36 ± 28.28	-18.71 ± 8.93	0.270^b^	0.791	0.274	0.773
	Pelvic tilt (mean ± SD)	14.38 ± 15.11	17.23 ± 14.0	11.09 ± 7.4	0.190^b^	0.799	0.648	0.184
Frontal plane spinal posture (Degrees)	Thoracic lateral tilt ((mean ± SD)	4.31 ± 2.99	5.0 ± 2.89	3.80 ± 2.37	0.384^a^	0.999	0.999	0.509
	Lumbar lateral tilt [median (IQR)]	7.50 (4.00)	4.00 (3.00)	4.00 (3.50)	**0.019**^c*^	0.006** G1 > G2	0.077	0.324
	Pelvic lateral tilt [median (IQR)]	6.00 (6.00)	4.00 (6.00)	6.00 (5.00)	0.691^c^	0.407	0.835	0.544
Sagittal plane spinal mobility (Degrees)	Thoracic mobility (Degrees) (mean ± SD)	18.55 ± 13.43	23.0 ± 11.21	20.47 ± 12.44	0.500^a^	0.731	0.999	0.999
	Lumbar mobility (Degrees) (mean ± SD)	79.0 ± 14.72	67.09 ± 25.58	67.09 ± 18.59	0.105^a^	0.188	0.197	0.999
	Hip/Sacral Mobility (Degrees) [Median (IQR)]	40.0 (17.0)	45.50 (29.0)	73.00 (37.00)	** < 0.001**^c*^	0.635	< 0.001** **G1 < G3**	< 0.001** **G2 < G3**
	Overall Sagittal Spinal Mobility (Degrees) (Mean ± SD)	142.05 ± 19.34	135.54 ± 22.16	157.90 ± 26.76	0.007^a*^	0.999	0.094	0.007** G2 < G3
Frontal plane spinal mobility (Degrees)	Thoracic mobility (Degrees) [median (IQR)]	24.50 (23.75)	25.00 (8.50)	36.00 (27.50)	0.031^c*^	0.845	0.028	0.017
	Lumbar mobility (Degrees) (mean ± SD)	27.09 ± 13.28	38.68 ± 13.04	34.42 ± 9.92	**0.009**^a*^	0.008** **G1 < G2**	0.160	0.774
	Sacral/hip mobility (Degrees) [median (IQR)]	14.50 (15.00)	9.00 (14.00)	20.00 (15.00)	**0.007**^c*^	0.309	0.038	0.002** **G2 < G3**
	Overall frontal spinal mobility (Degrees) [median (IQR)]	71.00 (41.75)	76.00 (42.00)	94.00 (40.50)	**0.005**^c*^	0.505	0.002** G1 < G3	0.014** G2 < G3

G1: pectus excavatum group, G2: pectus carinatum group, G3: healthy control groups, P0: comparison of all groups, P1: comparison of pectus excavatum and carinatum groups, P2: comparison of pectus excavatum and healthy control groups, P3: comparison of pectus carinatum and healthy control groups

*SD* standart deviation, *IQR* ınterquartile range, *n* number

^a^One-way ANOVA, ^b^Welch’s ANOVA, ^c^Kruskal Wallis test, *p < 0.050, **p < 0,016 (Bonferroni correction)

### Spinal posture

The thoracal kyphosis angle was different between groups (*p* < 0.001). The thoracic kyphosis angle in the PE and PC groups was higher than the control ones, respectively (*p* < 0.001; *p*:0.001). The lumbar lordosis and pelvic tilt angles were similar between groups (*p* > 0.05). The lumbar lateral tilt angle was different between groups (*p* < 0.019). The angle of lumbar lateral tilt was higher in the PE group compared to the PC (*p*:0.006). Thoracic and pelvic lateral tilt angles were not different (*p* > 0.05) (Table 3).

### Spinal mobility

In the sagittal plane, thoracic and lumbar mobility did not show significant differences between groups (*p* > 0.05); however, hip/sacral and overall sagittal mobility showed significant differences (*p* < 0.05). Hip/sacral mobility was lower in the PE and PC groups compared to the control group; respectively (*p* < 0.001; *p* < 0.001). Overall sagittal spinal mobility was lower in the PC group compared to the control group (*p*:0.007). In the frontal plane lumbar (*p*:0.009), sacral/hip (*p*:0.007), and overall frontal spinal mobility (*p*:0,005); but not for thoracic mobility (*p* > 0.05). The lumbar mobility in the frontal plane was lower in the PE groups compared to the PC groups (*p*:0.008). The hip/sacral mobility in the frontal plane was lower in the PC group compared to the control group (*p*:0.002). Overall frontal spinal mobility was lower in the PE and PC groups compared to the control group, respectively (*p*:0.002; *p*:0.014) (Table 3).

### Spinal position sense

The scores of the target angle test (repositing errors) referring to the spinal position sense of PE, PC, and control group were different (*p* < 0.001). The repositing errors of the PE and PC groups were higher than control groups, respectively (*p* < 0.001; *p*:0.014) (Table 3).

## Discussion

The current study was designed to investigate spinal posture, mobility, and position sense in PE and PC. It was observed that thoracic kyphosis and lumbar lateral tilt angles were higher in individuals with PE and PC compared to healthy controls. Additionally, hip/sacral mobility in the sagittal plane and overall frontal spinal mobility were lower in individuals with PE and PC compared to healthy controls. Moreover, hip/sacral mobility in the frontal plane and overall sagittal spinal mobility were lower in PC compared to healthy controls. The repositing error of spinal position sense of PE and PC was higher than healthy controls. Therefore, it can be inferred that adolescents with chest wall deformities (PE and PC) exhibited disorders of spinal alignment, reduced spinal mobility, and deterioration in spinal position sense.

Previous studies have highlighted postural changes in individuals with chest wall deformities [13, 14]. Steinman et al. reported that 94–95% of patients with PE and PC showed poorer posture compared to healthy individuals, based on physical examination findings [14]. Alaca et al. demonstrated that patients with PE and PC had poorer posture than healthy peers according to the New York Posture Scale. They reported that the incidence of postural disorders, such as forward head posture, increased kyphosis, and scoliosis, was higher among individuals with PE and PC [13]. The researchers highlighted that individuals with chest wall deformities tend to have poor posture, also known as “pectus posture”. This refers to the position that occurs due to the forward displacement of the shoulders and the development of thoracic kyphosis. However, they advised that further detailed three-dimensional biomechanical studies are required to explain this phenomenon more comprehensively [13]. Our study, which investigated spinal posture using a three-dimensional evaluation system, found that increased thoracic kyphosis and lumbar lateral tilt were indicative of scoliosis in individuals with PE and PC. Consistent with previous research, we also confirmed the presence of postural disorders in individuals with PE and PC. These findings may be attributed to the anatomical relationships between the chest wall and the spine. The change in the anatomical position of the chest wall structures may cause alterations in spinal alignment and biomechanics [7, 10, 12]. Furthermore, attempts to conceal the chest deformity in individuals with chest wall deformities may result in a deterioration in body image perception and a tendency to adopt a non-ideal posture [18, 19]. As a result, prolonged standing in a non-ideal posture may disrupt the normal biomechanics of the musculoskeletal system, subsequently leading to postural disorders [13, 14].

Our research was the first to explore the spinal mobility of adolescents with PE and PC. We found that the spinal mobility in both the sagittal and frontal planes was reduced in both PE and PC. Although no study has specifically investigated spinal mobility in individuals with PE and PC, a study examined the flexibility of trunk muscles, which indirectly measures the flexibility of the lower back and hamstring muscles using a sit-reach test. The study found that trunk flexibility was reduced in individuals with PE and PC compared to healthy peers [13]. The decreased spinal mobility observed in individuals with PE and PC may be associated with spinal alignment issues in this population. Studies conducted in different populations stated that spinal alignment problems may restrict spinal mobility [20, 21]. Wang et al. reported that osteoporotic patients with spinal alignment problems had less spinal mobility than those without [20]. A study conducted on adolescents and children found that those with obesity had poorer spinal posture as well as limited spinal mobility compared to those without obesity [21]. Therefore, spinal alignment disorders may limit spinal mobility in PE and PC.

We used the Spinal Mouse, a non-invasive three-dimensional method, to investigate the posture and mobility of the spine. It is a taken-for-granted knowledge that the X-ray is a gold standard evaluation method for posture and mobility of the spine [22]. However, clinicians and researchers are exploring new methods, such as the Spinal Mouse, to reduce the negative effects of radiation in cases requiring frequent radiological evaluation [23, 24]. Therefore, it may be a good alternative to evaluate the spinal posture and mobility of adolescents with PE and PC without posing any medical risks associated with radiation [24]. The Spinal Mouse provides information on the segmental and regional alignment and mobility of spine in sagittal and frontal planes. Regional results are obtained from the combination of information from each segment. These regional results can be used to analyze the angular values of scoliosis, kyphosis, or lordosis, which are common spinal disorders [24]. Since our study was a pioneering study in which the spinal spine was evaluated with Spinal Mouse in adolescents with chest deformity, we focused only on the regional evaluation results. Future studies in this field can also benefit from this technology by focusing on segmental evaluation.

The current study was the first to examine the spinal position sense of patients with PE and PC. We found that patients with PE and PC had weaker spinal position sense than healthy controls. Position sense is a key component of proprioception, which is the sense that enables the body to perceive the positions and movements of different body parts with each other and the external environment [25]. It can be adversely affected by various factors, including positional alignment faults such as postural disorders [25, 26]. The alignment of the spine plays a crucial role in supporting the proprioceptive system, which is responsible for maintaining proper body posture, and vice versa [25, 26]. It was previously reported that patients with spinal alignment disorders such as hyperkyphosis and scoliosis have a weaker sense of position [26, 27]. In addition to discovering weaker spinal position sense in individuals with PE and PC, we also observed that spinal alignment disorders, such as increased thoracic kyphosis and lumbar lateral tilt, are linked to scoliosis in PE and PC. Therefore, the deficiency in spinal position sense may be related to the positional faults in spinal alignment in individuals with PE and PC.

## Limitations

The main limitation of the current study is that it did not assess the posture and mobility of the cervical spine. This limitation arose because the spinal mouse device utilized in the study could not evaluate cervical spine posture and mobility. Consequently, further research should examine the posture and mobility of the cervical spine in individuals with chest wall deformities, as our investigation only focused on the thoracic, lumbar, and sacral spine. Secondly, although we had reached a determined sample size according to pre-power analysis, due to the public frequency of pectus deformities in adolescents [2, 3], further studies should focus on larger sample sizes than ours. The gender distribution in the study was not homogeneous. It is known that the incidence of chest deformities in males is approximately four times higher than in females [3]. In our study, the number of males was significantly higher than that of females. Future studies should also consider gender distribution.

## Conclusion

The current study was the first to explore spinal posture, mobility, and positional sense in adolescents with the most prevalent chest wall deformities, namely PE and PC. Given the close structural and biomechanical relationship between the chest wall and spine, it is essential to understand whether PE and PC influence spinal posture, mobility, and position sense. Our findings revealed that adolescents with PE and PC exhibited reduced spinal mobility, spinal alignment disorders, and deteriorated spinal position sense compared to their healthy peers.

The findings provide valuable insights into the characteristics of the spine in adolescents with chest deformities and the unique mechanical challenges associated with these deformities. By addressing spinal evaluation in pectus deformities and confirming spinal alignment disorders like increased thoracic kyphosis and scoliosis, we are the first to highlight the information that spinal mobility and proprioception disorders can also occur in adolescents with chest deformities. It is well known that spinal disorders can lead to early musculoskeletal pathologies and pain, and this can be observed even in adolescence [28, 29]. Our findings indicate that adolescents with chest deformities have a higher possibility of experiencing spinal alignment, mobility, and proprioception disorders compared to adolescents without chest deformities. Therefore, it is clinically important to manage these issues. In managing chest deformities in adolescents, pediatric surgeons and physical therapists need to collaborate in addressing spinal alignment, mobility, and proprioception disorders through an appropriate rehabilitation program. Based on the data from the present study, we believe that not only the deformity itself should be evaluated, but also the structure and function of the spine should be evaluated and treated with a multi-disciplinary approach in adolescents with chest deformities.

## Data Availability

No datasets were generated or analyzed during the current study.
